# Transcription Factors Efg1 and Bcr1 Regulate Biofilm Formation and Virulence during *Candida albicans*-Associated Denture Stomatitis

**DOI:** 10.1371/journal.pone.0159692

**Published:** 2016-07-25

**Authors:** Junko Yano, Alika Yu, Paul L. Fidel, Mairi C. Noverr

**Affiliations:** 1 Department of Oral and Craniofacial Biology, Dental School, Louisiana State University Health Sciences Center, New Orleans, Louisiana, United States of America; 2 Department of Prosthodontics, Dental School, Louisiana State University Health Sciences Center, New Orleans, Louisiana, United States of America; King's College London Dental Institute, UNITED KINGDOM

## Abstract

Denture stomatitis (DS) is characterized by inflammation of the oral mucosa in direct contact with dentures and affects a significant number of otherwise healthy denture wearers. The disease is caused by *Candida albicans*, which readily colonizes and form biofilms on denture materials. While evidence for biofilms on abiotic and biotic surfaces initiating *Candida* infections is accumulating, a role for biofilms in DS remains unclear. Using an established model of DS in immunocompetent animals, the purpose of this study was to determine the role of biofilm formation in mucosal damage during pathogenesis using *C*. *albicans* or mutants defective in morphogenesis (*efg1*^*-/-*^) or biofilm formation (*bcr1*^*-/-*^*)*. For *in vivo* analyses, rats fitted with custom dentures, consisting of fixed and removable parts, were inoculated with wild-type *C*. *albicans*, mutants or reconstituted strains and monitored weekly for fungal burden (denture and palate), body weight and tissue damage (LDH) for up to 8 weeks. *C*. *albicans* wild-type and reconstituted mutants formed biofilms on dentures and palatal tissues under *in vitro*, *ex vivo* and *in vivo* conditions as indicated by microscopy demonstrating robust biofilm architecture and extracellular matrix (ECM). In contrast, both *efg1*^*-/-*^ and *bcr1*^*-/-*^ mutants exhibited poor biofilm growth with little to no ECM. In addition, quantification of fungal burden showed reduced colonization throughout the infection period on dentures and palates of rats inoculated with *efg1*^*-/-*^, but not *bcr1*^-/-^, compared to controls. Finally, rats inoculated with *efg1*^*-/-*^ and *bcr1*^*-/-*^ mutants had minimal palatal tissue damage/weight loss while those inoculated with wild-type or reconstituted mutants showed evidence of tissue damage and exhibited stunted weight gain. These data suggest that biofilm formation is associated with tissue damage during DS and that Efg1 and Bcr1, both central regulators of virulence in *C*. *albicans*, have pivotal roles in pathogenesis of DS.

## Introduction

Denture stomatitis (DS) is an inflammatory fungal infection, presenting as erythematous inflammation beneath primarily maxillary (upper) dentures [1–7]. DS is the most common form for oral candidiasis, affecting approximately 30–75% of otherwise healthy denture wearers. *Candida albicans* being the most common etiologic agent; however non-albicans *Candida* species are also associated with infection [8]. Symptoms of *Candida*-associated denture stomatitis range from mild to severe, including palatal edema, painful inflammation, and papillary hyperplasia (small pebble-like sores) [9]. Denture stomatitis can have a negative impact on the quality of life for those affected, with very high recurrence rates despite treatment with antifungal therapy [10–15]. Despite its prevalence, the pathogenesis of DS and role of fungal virulence factors contributing to infection are not well understood.

*C*. *albicans* readily forms biofilms on denture material in vitro and in vivo, which consist of a network of hyphae and yeast encased in a polysaccharide matrix [16–19]. *C*. *albicans* biofilms exhibit increased resistance to drugs and antifungal host defenses [20–23]. It has been proposed that the ability to form biofilms is a key requirement in the pathogenesis of denture stomatitis. However, this has not been directly examined clinically, or in a clinically relevant model of DS. Our laboratory has developed a clinically relevant animal model of DS using immunocompetent rats [24]. Rats are fitted with an innovative rodent denture system that uses individual impression casting for production of custom fitted devices made from modern denture material. This denture system was engineered with both fixed and removable portions to facilitate longitudinal studies (patent #8753113). Using this system, we developed a model of *Candida*-associated denture stomatitis in immunocompetent rats, which results in palatal inflammation and clinical signs of disease (palate erythema and edema) following both denture and palate biofilm formation [25]. In this study, we are extending our observations to examine the requirement for biofilm formation in our DS model. We tested mutants in master regulators of morphogenesis (Efg1) and biofilm formation (Bcr1), which have previously been shown to form poor or no biofilms on abiotic or biotic surfaces [26–29].

## Materials and Methods

### Animals

Male hairless euthymic rats (400-600g) were purchased from Charles River Laboratories. All rats were maintained at an American Association for the Accreditation of Laboratory Animal Care (AAALAC)-accredited animal facility at Louisiana State University Health Sciences Center (LSUHSC) and housed in accordance with the procedures outlined in the Guide for the Care and Use of Laboratory Animals under a protocol proposal approved by the LSUHSC Institutional Animal Care and Use Committee. The animals were fed pellet rodent food during the initial acclimation period and then weaned onto gel diet 76A (Clear H2O, Westbrook, ME) 1 week prior to denture installation. The animals were maintained on the gel diet for the remainder of the study to minimize accumulation of food debris on the denture. For euthanasia, rats will be exposed to CO_2_ followed by a pneumothorax with an open chest cavity to ensure death. This method is consistent with the recommendation of the Panel on Euthanasia of the American Veterinarian Medical Association.

### Human Saliva Collection

Saliva was obtained from banked specimens de-identified from normal healthy volunteers enrolled in a previous study that was conducted in accordance with the stated guidelines of the Institutional Review Board of Louisiana State University Health Sciences Center. Saliva was obtained by collecting unstimulated saliva expelled into a 50ml conical tube. The sterile filtered saliva was aliquoted into 1-ml volumes and stored frozen at −80°C until use. Samples were pooled for use in the present study.

### *Candida albicans* strains and handling

*C*. *albicans* strain DAY185 is a prototrophic control strain derived from a triple auxotrophic strain (BWP17; parent, SC5314) and is commonly used to produce knockout and complemented stains of *C*. *albicans* [30]. Strains HLC52 (*efg1/efg1*), HLC74 (*efg1/efg1 + EFG1*) were kindly provided by G. R. Fink (Whitehead Institute, Cambridge MA). Strains CJN702 (*bcr1*/*bcr1*) and CJN698 (*bcr1*/*bcr1 + pBCR1*) were a gift from A. P. Mitchell (Carnegie Mellon University, Pittsburgh, PA). Frozen stocks of fungal strains were kept at -80°C and streaked on to Sabouraud dextrose agar (SDA) (BD) or CHROMagar^™^ Candida (CHROMagar, Paris, France). A single colony was transferred to 20 ml of yeast extract-peptone-dextrose (YPD) broth and incubated with shaking at 30°C for 18 h (stationary phase). To determine fungal concentrations, all isolates were washed 3 times by centrifugation in sterile phosphate-buffered saline (PBS), counted on a hemocytometer, and diluted in sterile PBS to the desired inocula.

### Quantitative Culture Assay

To enumerate fungal burden, samples were serially diluted by 10-fold in sterile 1X PBS and cultured on SDA plates at 37°C overnight. Resulting colonies were counted visually and expressed as colony forming units (CFU’s).

### In Vitro Model of Denture Biofilm Formation

Polymethyl methacrylate (PMMA) acrylic resin (Self curing denture; Lang Dental, Wheeling, Il) was prepared according to the manufacturer’s instructions and spread onto glass microscope slides. The PMMA was scored to make uniform sized squares and allowed to cure. Denture chips were placed into sterile 6 or 12 well plates. For inoculation, *C*. *albicans* wild-type DAY185, mutants or reconstituted strains were diluted in sterile 1X PBS to a final concentration of 1 x 10^7^ CFU/ml. For inoculation, 100 μl of *C*. *albicans* suspension was added to the surface of the denture chip. The sample was incubated at 37°C for 3 h then rinsed gently with 1X PBS and submerged in saliva for an additional 24 h at 37°C before being processed for microscopic analysis.

### Ex vivo Model of Mucosal Biofilm Formation

Rats were euthanized and palatal tissue was excised using a sterile scalpel. Palate tissue was placed into sterile 12 well plates containing sterile 1X PBS. Tissues were not submerged and the epithelial surface remained air-exposed. For inoculation, *C*. *albicans* wild-type DAY185, mutants or reconstituted strains were diluted in sterile 1X PBS to a final concentration of 1 x 10^7^ CFU/ml. For inoculation, 100 μl of *C*. *albicans* suspension was added to the surface of the palate tissue. The sample was incubated at 37°C for 3 h then rinsed gently with 1X PBS and submerged in 1X PBS for an additional 24 h at 37°C before being processed for microscopic analysis.

### In Vivo Rat Denture Stomatitis Model

All animals were housed and handled according to institutionally recommended guidelines. All animal protocols were reviewed and approved by the Institutional Animal Care and Use Committee (IACUC) of the LSU Health Sciences Center New Orleans. Each rat was housed separately in an individual cage throughout the study period. For in vivo experiments, we used our custom-fitted denture system in rats (patent # 8753113) [31]. This innovative rodent denture system was engineered with both fixed and removable portions. For custom fitting, impressions were taken from individual rats, using light body VPS impression material (Aquasil Ultra LC, Dentsply Caulk) applied to the maxillary palate. Impressions were used to produce stone mold templates for production of the fixed and removable dentures. For installation, rats were first anesthetized by intraperitoneal injection with 90 mg/kg ketamine + 10 mg/kg xylazine. The rats remained anesthetized for approximately one hour to complete the installation process. The fixed portion of the denture, which is embedded with nickel magnets, is anchored to the rear molars of the rat by orthodontic wires. The removable portion, which fits over the anterior palate, attaches magnetically to the fixed portion via an embedded metal rod. The removable portion can easily be detached for sampling and replaced, which allows for longitudinal analyses.

Prior to inoculation, rats were weaned onto a gel diet (ClearH2O) and amoxicillin/clavulanic acid (Clavamox^®^, Pfizer, NY) (31.25 mg in 200 ml) was added to the drinking water for 4 days to reduce oral bacteria. For inoculation, rats were anesthetized with continuous administration of isoflurane via nose cone. Rats were inoculated with 1 x 10^9^ CFU *C*. *albicans* wild-type DAY185, mutants or reconstituted strains in oral gel formulation (semisolidified with 5% carboxymethylcellulose in PBS, Sigma), which was applied to the palate underneath the removable denture. The rats remained anesthetized until the removable denture was securely reinstalled with the gel inoculum in place, for approximately 5 minutes. Inoculation was performed a total of 3 times with 3-day intervals, and rats were monitored weekly for oral evaluation and weight measurements.

### Microbial Burden

For quantification of fungal burden on the denture and palate tissue, animals were anesthetized with 3% isoflurane via inhalation. The removable portion of the denture was detached and the intaglio surface of the denture and the palate were each swabbed with individual sterile cotton tipped applicators. Swabbing was performed by gently sliding the cotton applicator on the surface of the hard palate and along the ridges of the rugae. The applicators were vortexed in 200 of 1X μl PBS and fungal burden was analyzed by quantitative culture assay. Rats were evaluated for denture and palatal fungal burden weekly throughout the study period. For microscopy, palate tissue was excised and dentures were removed from euthanized rats at designated time points. Controls consisted of naïve animals and uninoculated animals with the denture system.

### Lactose dehydrogenase (LDH) Assay

Levels of LDH release detected in palates were determined colorimetrically by LDH assay kit as manufacturer’s instructions (Abcam, Cambridge, UK). The activity of LDH in the supernatants of palate swab suspensions was measured by recording the rate of change in NADH concentrations after interaction with a colorimetric probe. The absorbance was read at a wavelength of 450 nm using a Multiskan Ascent microplate photometer (Labsystems, Helsinki, Finland). The results were expressed as optical density at 450 nm (OD_450_).

### Confocal Microscopy (CM)

Excised palate tissue or dentures were fixed in 10% formalin overnight at 4°C and stored until use. Samples were rinsed twice in 1X PBS and stained for 20 min at room temperature with 1 mg/ml Calcofluor White (Fluka), which stains the fungal cell wall, and 50 ug/ml Concanavalin A-Texas Red conjugate (Con A-TR, Molecular Probes), which is a lectin that stains the biofilm extracellular matrix. Tissue or denture material was removed from staining wells, gently rinsed with 1x PBS to remove exogenous stain, and placed onto glass microscope slides. Images were captured using a 60X objective on an Olympus FV100 confocal microscope. Quantification of biofilm thickness was assessed by measuring five cross-sectional depths of biofilms using the Fluoview software and averaged per image.

### Scanning Electron Microscopy (SEM)

Excised palate tissue or dentures were fixed in 10% formalin overnight at 4°C and stored until use. Samples were rinsed twice in 1x PBS before post fixing in 2% osmium tetroxide for 30 min at room temperature. Following two washes in 1X PBS the samples were dehydrated using the following reagents and incubation times: 20% ethanol, 1 min; 40% ethanol, 1 min; 60% ethanol, 1 min; 80% ethanol, 1 min; 100% ethanol, 3 min; 100% acetone, 3 min. Samples were then air-dried overnight and loaded onto SEM studs with double-sided magnetic tape and carbon-coated. Specimens were viewed at 100-1000x magnifications on a Hitachi S-2700 SEM (LSUHSC Imaging Core) with the voltage set to 15kV.

### Statistics

All experiments used groups of 2 to 5 rats and were independently repeated, except where noted. Statistical comparisons of biofilm cross-sectional thickness between groups were analyzed using a one-way analysis of variance (ANOVA) followed by the Tukey’s post hoc multiple comparison test. Analyses of fungal burden and body weight were performed using repeated measures ANOVA to identify changes over time in each group. The Student’s *t* test was used to compare the experimental groups to the positive control for specific time-points (weeks). For LDH analyses the non-parametric Kruskal-Wallis test followed by Mann-Whitney U post hoc tests were used to analyze data that was no normally distributed. Significant differences were defined at a confidence level where *P* is <0.05. All statistical analyses were performed using Prism software (Graph Pad, San Diego, CA).

## Results

### *EFG1* and *BCR1* are required for *C*. *albicans* biofilm formation on denture material and palate tissue in vitro

**Fig 1:**
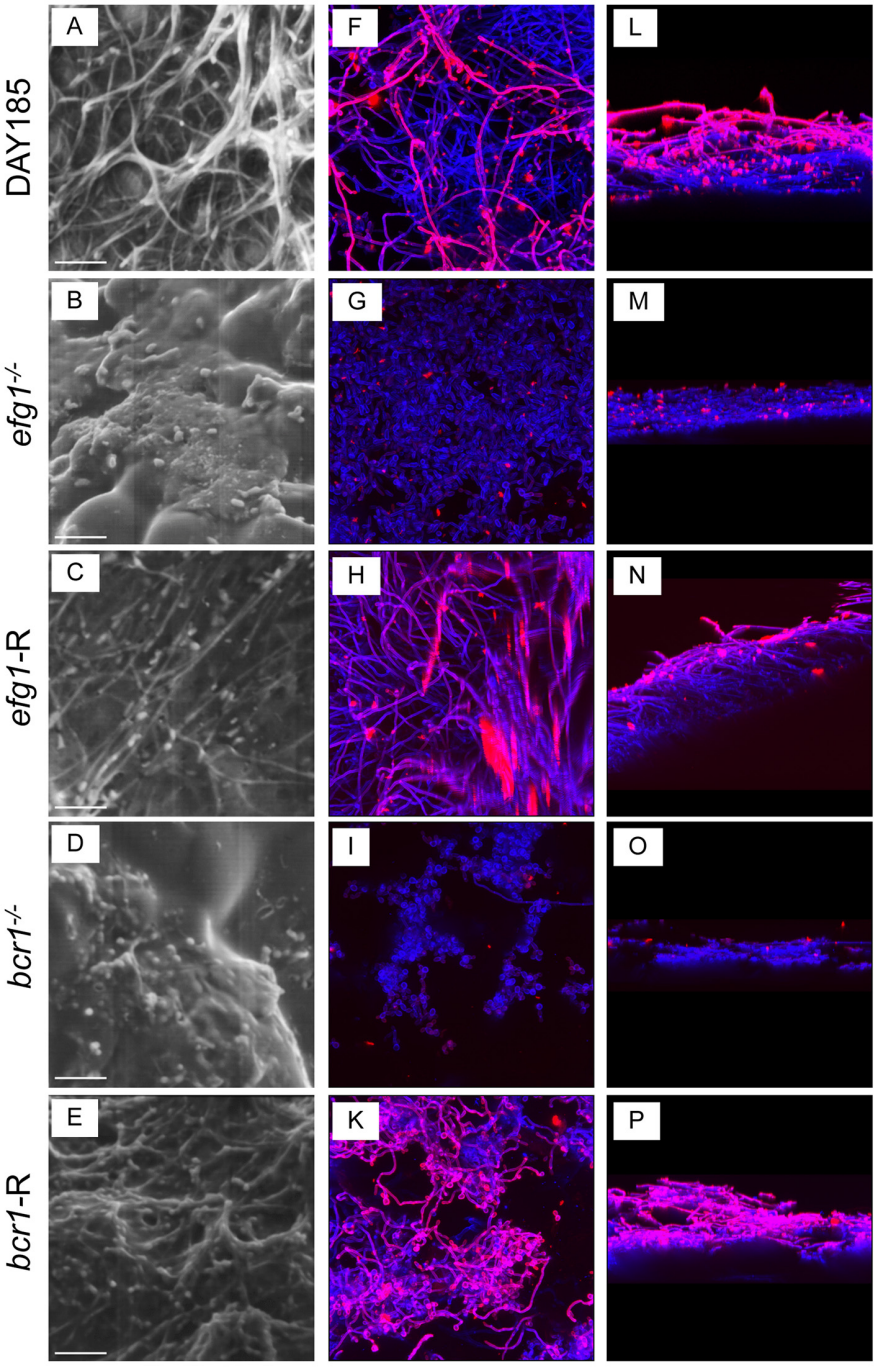
*In vitro* biofilm formation on denture materials in human saliva. Pre-sterilized denture material was inoculated with 1 x 10^6^ *C*. *albicans* DAY185 (A, F, K), *efg1*^*-/-*^ (B, G, L), *efg1*-reconstituted (C, H, M), *bcr1*^*-/-*^ (D, I, N) or *bcr1*-reconstituted (E, J, O) strain and incubated in saliva for 24h at 37°C to allow biofilm growth. The denture materials were then processed for SEM (A-E) or stained with calcofluor white (blue; stains fungal chitin in the cell wall) or Concanavalin A-Texas Red conjugate (red; stains mannose in the cell wall and ECM) and examined by fluorescent confocal microscopy to visualize biofilms in XY (F-J) and XYZ (K-O) views. Each panel shows a representative image of 3 repeats. Scale bar = 50 μm.

**Fig 2:**
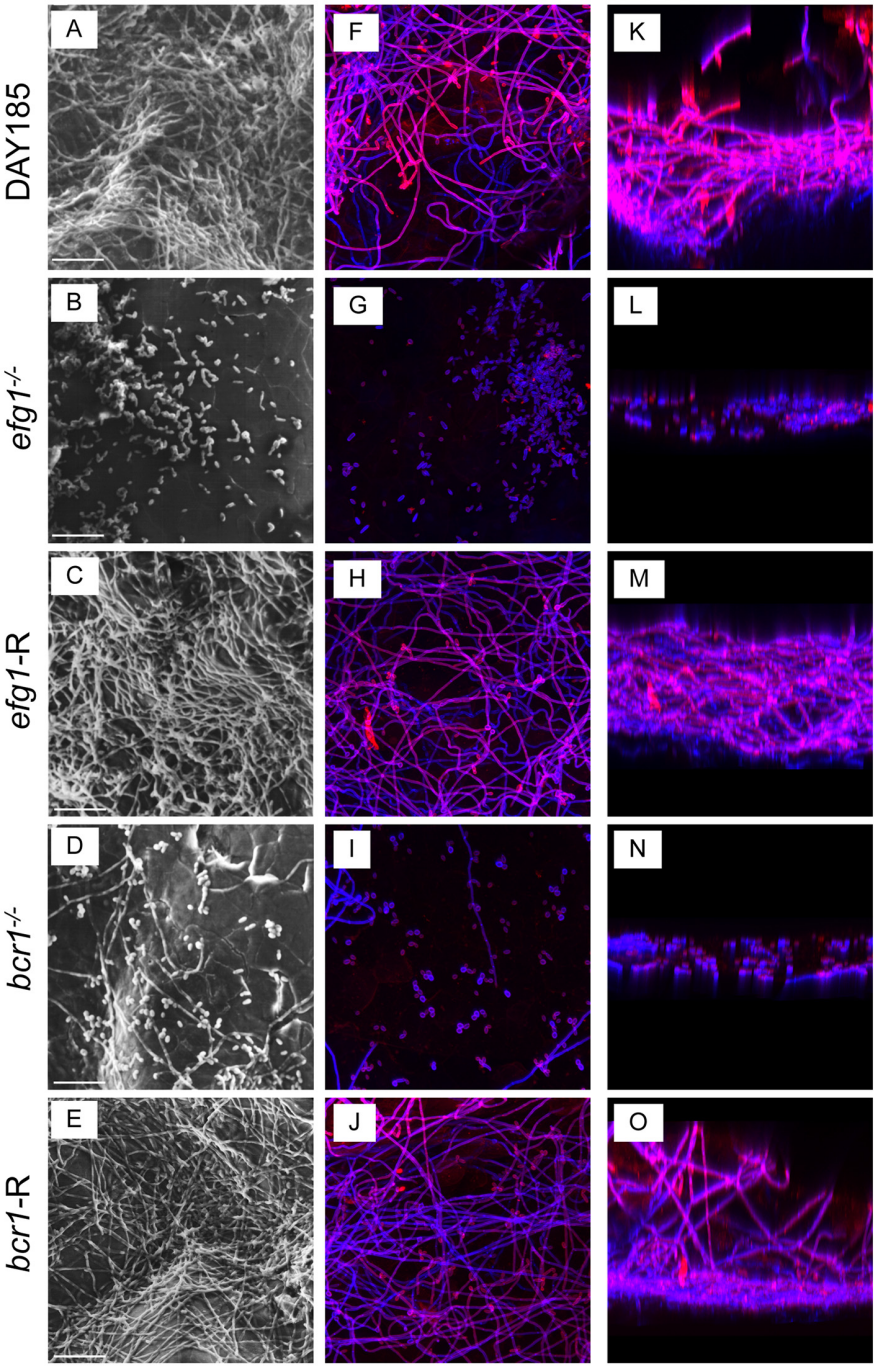
*Ex vivo* biofilm formation on rat palate tissue. Excised rat palate tissues were inoculated with 1 x 10^6^ *C*. *albicans* DAY185 (A, F, K), *efg1*^*-/-*^ (B, G, L), *efg1*-reconstituted (C, H, M), *bcr1*^*-/-*^ (D, I, N) or *bcr1*-reconstituted (E, J, O) strain and incubated in PBS for 24h at 37°C to allow biofilm growth. The tissues were then processed for SEM (A-E) or stained with calcofluor white (blue; stains fungal chitin in the cell wall) or Concanavalin A-Texas Red conjugate (red; stains mannose in the cell wall and ECM) and examined by fluorescent confocal microscopy to visualize biofilms in XY (F-J) and XYZ (K-O) views. Each panel shows a representative image of 2 repeats. Scale bar = 50 μm.

We evaluated *C*. *albicans* mutant strains with deletions in transcriptional regulators of morphogenesis and other co-regulated virulence traits (*efg1*^-/-^) and biofilm formation (*bcr1*^-/-^*)* using in vitro and ex vivo models. For the in vitro model, PMMA denture samples were inoculated with *C*. *albicans* strains in filter-sterilized human saliva and allowed to grow for 24h at 37°C to mimic in vivo conditions. Wild-type (WT) *C*. *albicans* strain DAY185 exhibited typical biofilm architecture on denture material by SEM and confocal analysis (Fig 1), with an extensive hyphal network and mannose (red) staining in the upper biofilm layers, a component of the cell wall and extracellular matrix (ECM). On palate tissue explants, WT *C*. *albicans* produced a dense biofilm and ECM, and appeared coated in extracellular material by SEM (Fig 2). This is in stark contrast to both the efg1^-/-^ and *bcr1*^-/-^ mutants on either denture material or palate explants, which formed no or very few hyphae and little ECM. These defects were rescued in the complemented strains (Figs 1 and 2).

### *EFG1* and *BCR1* are required for *C*. *albicans* biofilm formation on denture material and palate tissue in vivo

**Fig 3:**
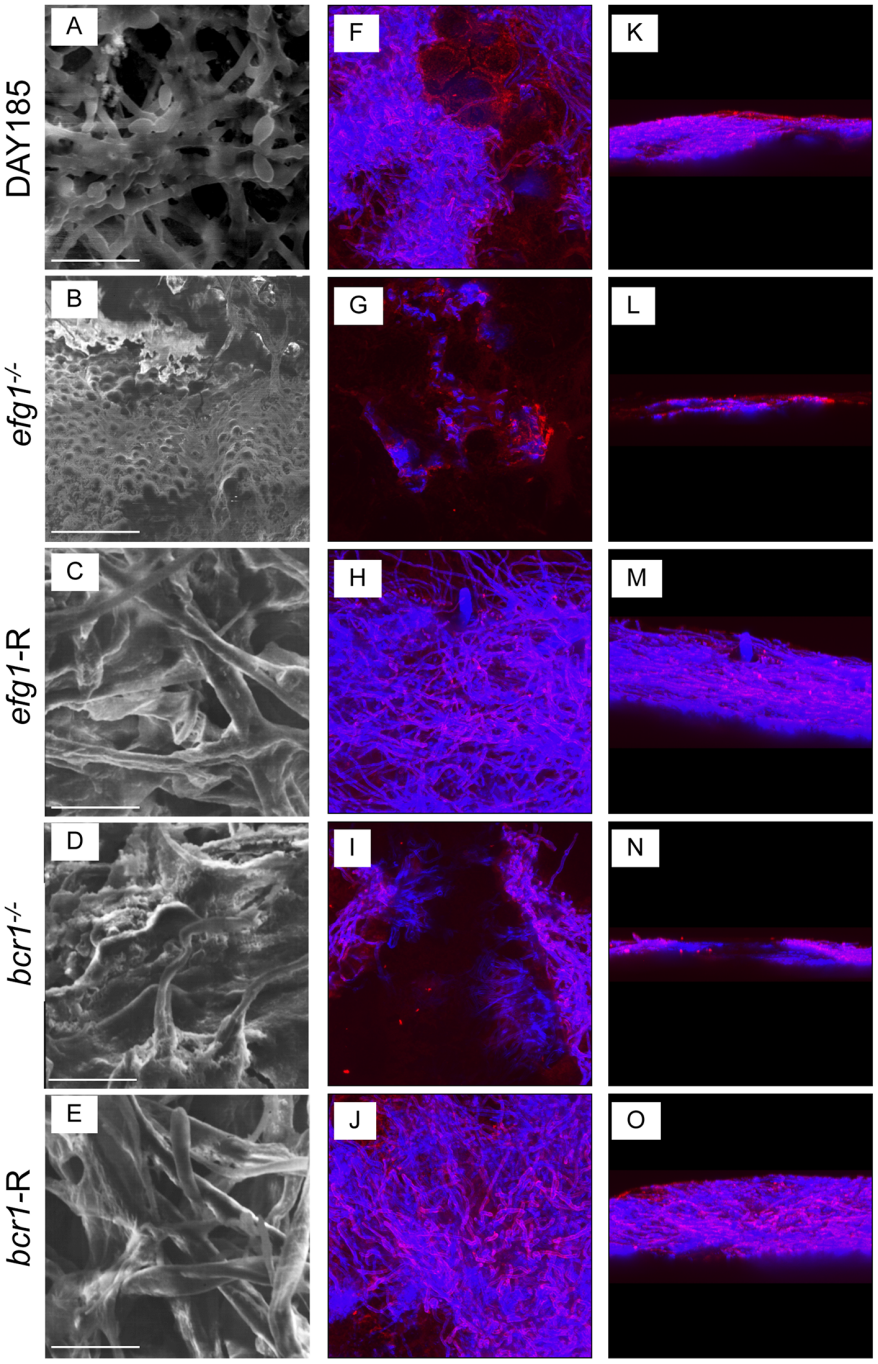
The role of *C*. *albicans* EFG1 and BCR1 in biofilm formation on dentures *in vivo*. Equilibrated rats (n = 4/group) were weaned onto gel diet and fitted with dentures. Rats were given broad-spectrum antibiotics in the drinking water for 4 days prior to inoculation. Rats were inoculated 3x at 3-day intervals with 1x10^9^ CFU *C*. *albicans* DAY185 (A, F, K), *efg1*^*-/-*^ (B, G, L), *efg1*-reconstituted (C, H, M), *bcr1*^*-/-*^ (D, I, N) or *bcr1*-reconstituted (E, J, O) strain. Dentures were removed from inoculated rats at 4 weeks post-inoculation. The dentures were processed for SEM (A-E) or stained with calcofluor white (blue; stains fungal chitin in the cell wall) or Concanavalin A-Texas Red conjugate (red; stains mannose in the cell wall and ECM) and examined by fluorescent confocal microscopy to visualize biofilms in XY (F-J) and XYZ (K-O) views. Each panel shows a representative image of 2–3 animals. Scale bar = 50 μm.

**Fig 4:**
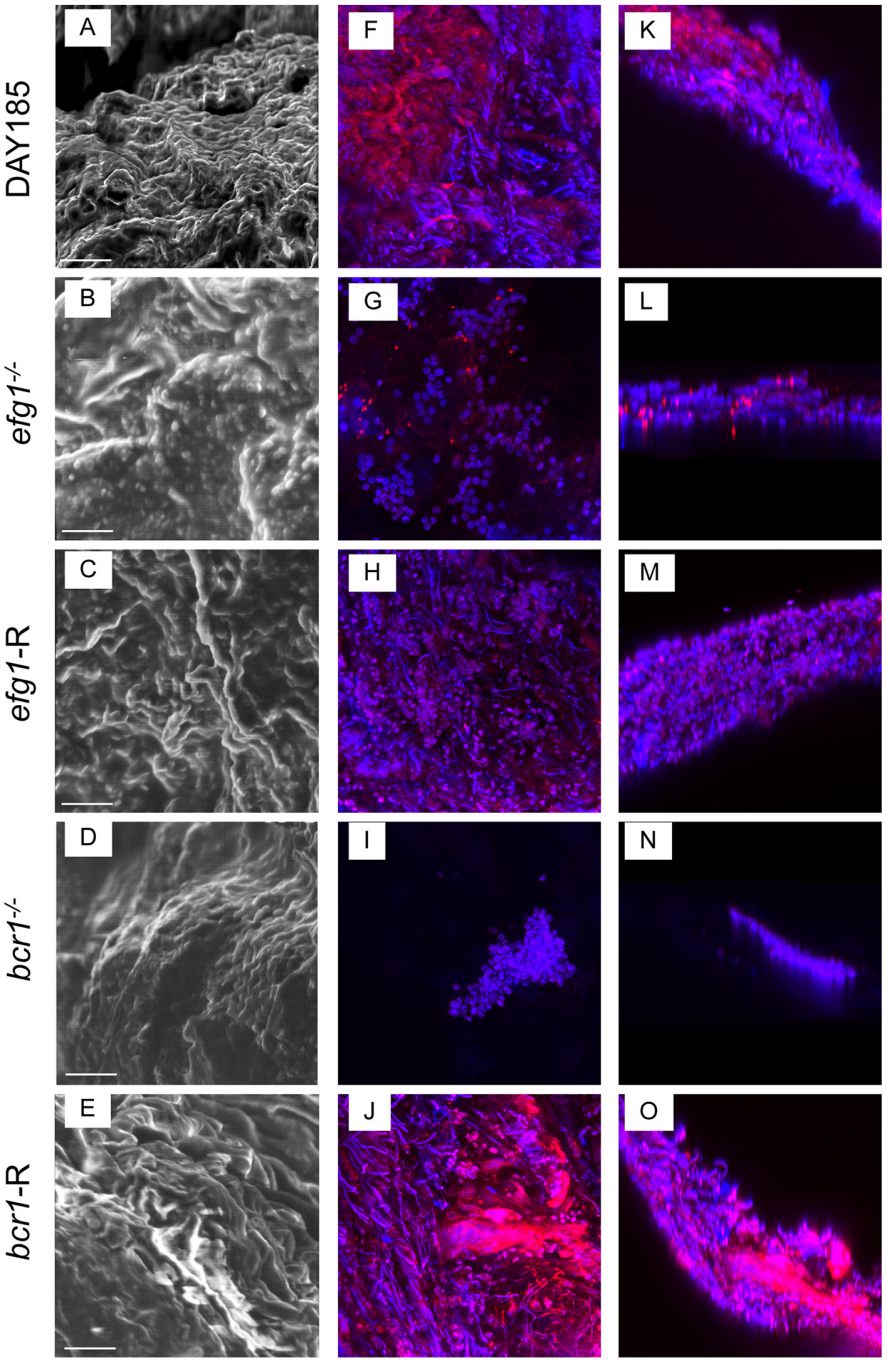
The role of *C*. *albicans* EFG1 and BCR1 in biofilm formation on palate tissue *in vivo*. Equilibrated rats (n = 4/group) were weaned onto gel diet and fitted with dentures. Rats were given broad-spectrum antibiotics in the drinking water for 4 days prior to inoculation. Rats were inoculated 3x at 3-day intervals with 1x10^9^ CFU *C*. *albicans* DAY185 (A, F, K), *efg1*^*-/-*^ (B, G, L), *efg1*-reconstituted (C, H, M), *bcr1*^*-/-*^ (D, I, N) or *bcr1*-reconstituted (E, J, O) strain. Palate tissues were excised from inoculated rats at 4 weeks post-inoculation. The tissue samples were then processed for SEM (A-E) or stained with calcofluor white (blue; *C*. *albicans*) or Concanavalin A-Texas Red conjugate (red; ECM) and examined by fluorescent confocal microscopy to visualize biofilms in XY (F-J) and XYZ (K-O) views. Each panel shows a representative image of 2–3 animals. Scale bar = 50 μm.

**Fig 5:**
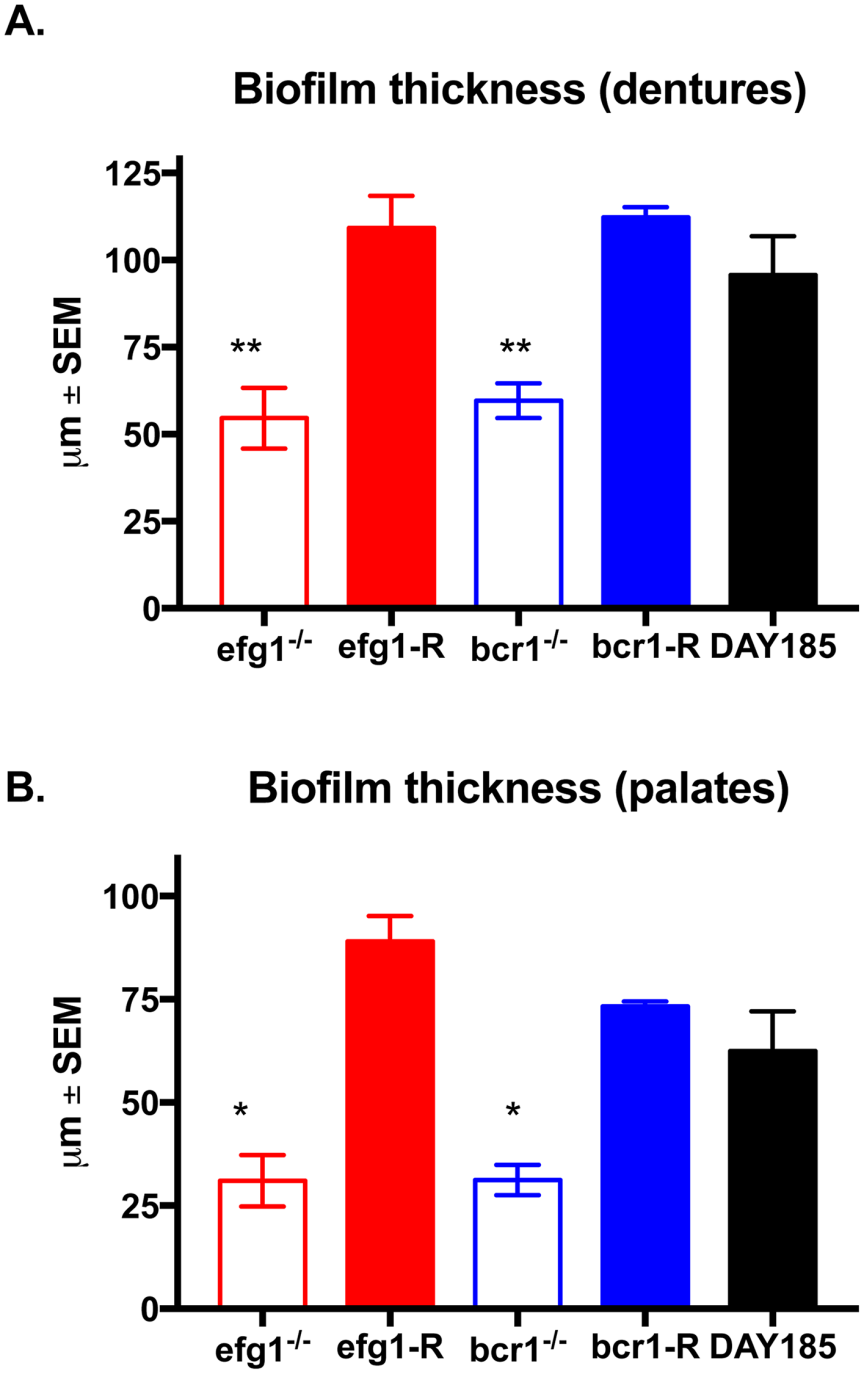
Quantification of biofilm thickness on dentures and palate tissue *in vivo*. Equilibrated rats were weaned onto gel diet and fitted with dentures. Rats were given broad-spectrum antibiotics in the drinking water for 4 days prior to inoculation. Rats were inoculated 3x at 3-day intervals with 1 x 10^9^ *C*. *albicans* DAY185, *efg1*^*-/-*^ or *bcr1*^*-/-*^ strain. (A) Dentures and (B) palate tissues were removed from inoculated rats at 4 weeks post-inoculation. Samples were stained with calcofluor white (blue; stains fungal chitin in the cell wall) or Concanavalin A-Texas Red conjugate (red; stains mannose in the cell wall and ECM) and examined by fluorescent confocal microscopy to visualize biofilms. Cross-sectional images of biofilms were visualized by confocal microscopy at 600X magnification, and the depths of biofilms were measured using the Fluoview software. Figure represents cumulative results from 2 independent experiments with 2–3 animals per group and assessment of 5 random areas per animal. Data were analyzed using a one-way ANOVA followed by the Tukey’s post hoc multiple comparison test. *, *P* < 0.05; **, *P* < 0.01 compared to the WT control.

We next evaluated the ability of transcription factor deficient mutants to form biofilms on denture material and palate tissue in our rat model of denture stomatitis. This model uses custom-fitted fixed and removable dentures that are in direct contact with the intaglio surface of the rat palate tissue in immunocompetent animals. Rats were inoculated and analyzed at 4 weeks post-inoculation, a time point in which mature biofilms are present in vivo. While WT strain DAY185 showed typical thick biofilm architecture on the denture and some visible ECM, the efg1^-/-^ mutant remained in the yeast form with sparse thin growth patterns (Fig 3). Although the bcr1^-/-^ mutant formed hyphae, the resulting biofilm was significantly thinner by cross-sectional view and growth patterns were sparse. The reconstituted strains, on the other hand, showed confluent biofilm architecture and morphology similar to the WT strain. In contrast to the in vivo biofilms on the dentures, WT strain DAY185 produced significantly more ECM on palatal tissue evident in SEM and confocal micrographs (Fig 4), and there was a more obvious layer of yeast attached to the epithelium with a hyphal network composing the upper layers of the biofilm. Both the efg1^-/-^ and bcr1^-/-^ strains lacked hyphal growth, and displayed little to no ECM staining by confocal microscopy. Both reconstituted mutants appeared similar to WT *C*. *albicans*, with the exception of the efg1-R, which appeared to produce less ECM (Fig 4). Quantitative assessment of biofilm thickness confirmed the microscopic imaging; efg^-/-^ and bcr1^-/-^ had significantly reduced thickness compared to the WT strain, whereas the reconstituted strains were similar in thickness to the WT strain (Fig 5).

### *EFG1* is required for optimal colonization of the denture and palate in vivo

**Fig 6:**
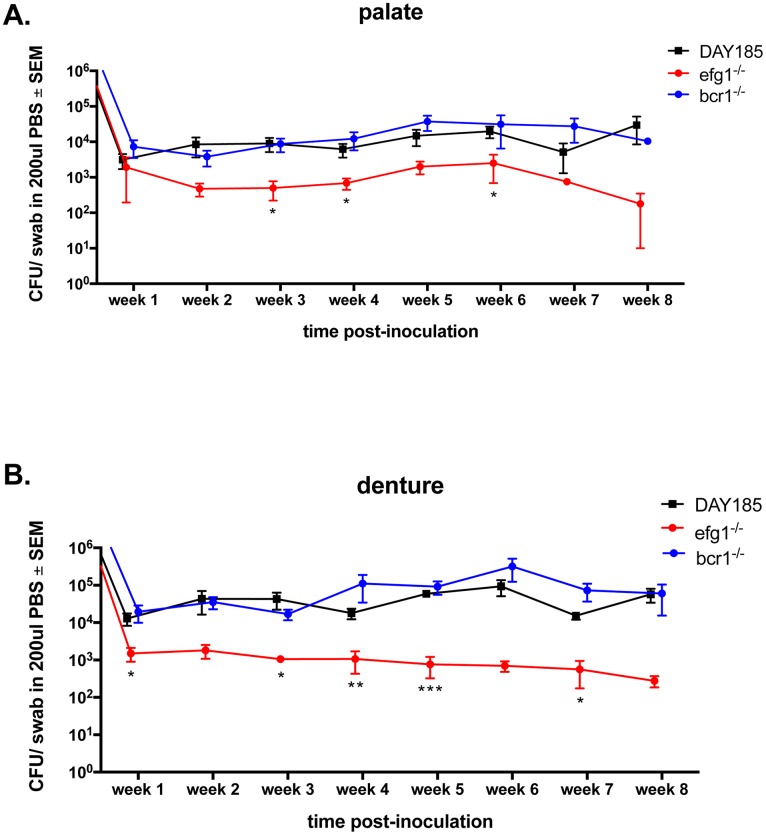
Fungal colonization levels on dentures and palate tissue in rats inoculated with *C*. *albicans* biofilm deficient strains. Equilibrated rats were weaned onto gel diet and fitted with dentures. Rats were given broad-spectrum antibiotics in the drinking water for 4 days prior to inoculation. Rats were inoculated 3x at 3-day intervals with 1 x 10^9^ *C*. *albicans* DAY185, *efg1*^*-/-*^ or *bcr1*^*-/-*^ strain. Swab samples of both the palate (A) and denture (B) were taken weekly for a period of 8 weeks post-inoculation. Fungal burdens were assessed from swab suspension fluid. Figure represents cumulative results from 2 independent experiments with 4–5 animals per group. Data were analyzed using Repeated Measures ANOVA (longitudinal data for each group) and the unpaired Student’s *t* test (individual time points (weeks), experimental vs. control). *, *P* < 0.05; **, *P* < 0.01; ***, *P* < 0.001.

Because our denture system is composed of both fixed and removable portions, we are able to monitor fungal colonization longitudinally. Rats inoculated with the bcr1^-/-^ mutant or the reconstituted strains showed similar levels of colonization compared to the WT *C*. *albicans* strain for up to 8 weeks post-inoculation on both the denture (Fig 6A) and palate (Fig 6B). Conversely, the efg1^-/-^ mutant exhibited significantly reduced colonization of both the palate and denture compared to the WT strain, with up to 1.5 log less fungal burden over time. Repeated measures analysis showed that each group maintained similar levels of colonization throughout the 8 week period (P > 0.05).

### *EFG1* and *BCR1* are required for virulence during denture stomatitis

**Fig 7:**
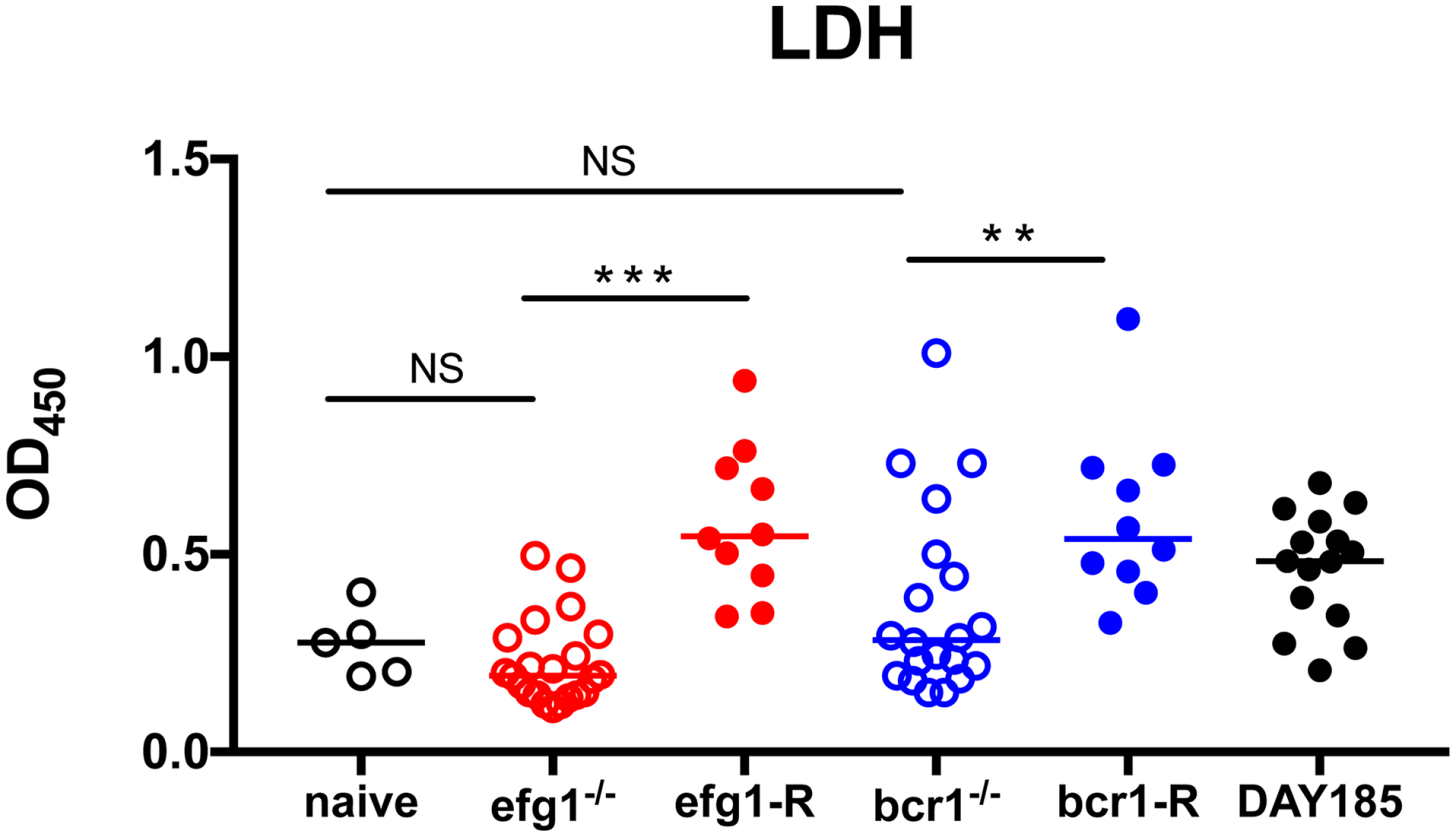
Mucosal damage in rats fitted with dentures inoculated with *C*. *albicans* biofilm deficient strains. Equilibrated rats were fitted with dentures, weaned onto gel diet, given antibiotics in the drinking water, and inoculated 3 times at 3 day intervals with 1 x 10^9^ *C*. *albicans* DAY185, *efg1*^*-/-*^ or *bcr1*^*-/-*^ or reconstituted mutants. Swab samples of the palate were taken weekly for a period of 4 weeks post-inoculation. LDH levels were assessed from swab suspension fluid. The results represent cumulative data from 2 independent experiments at 1–4 weeks post-inoculation with 4–5 animals per group. Data were analyzed using the Kruskal-Wallis test followed by the post hoc Mann-Whitney U test. n.s., not significant; **, *P* < 0.01; ***, *P* < 0.001.

**Fig 8:**
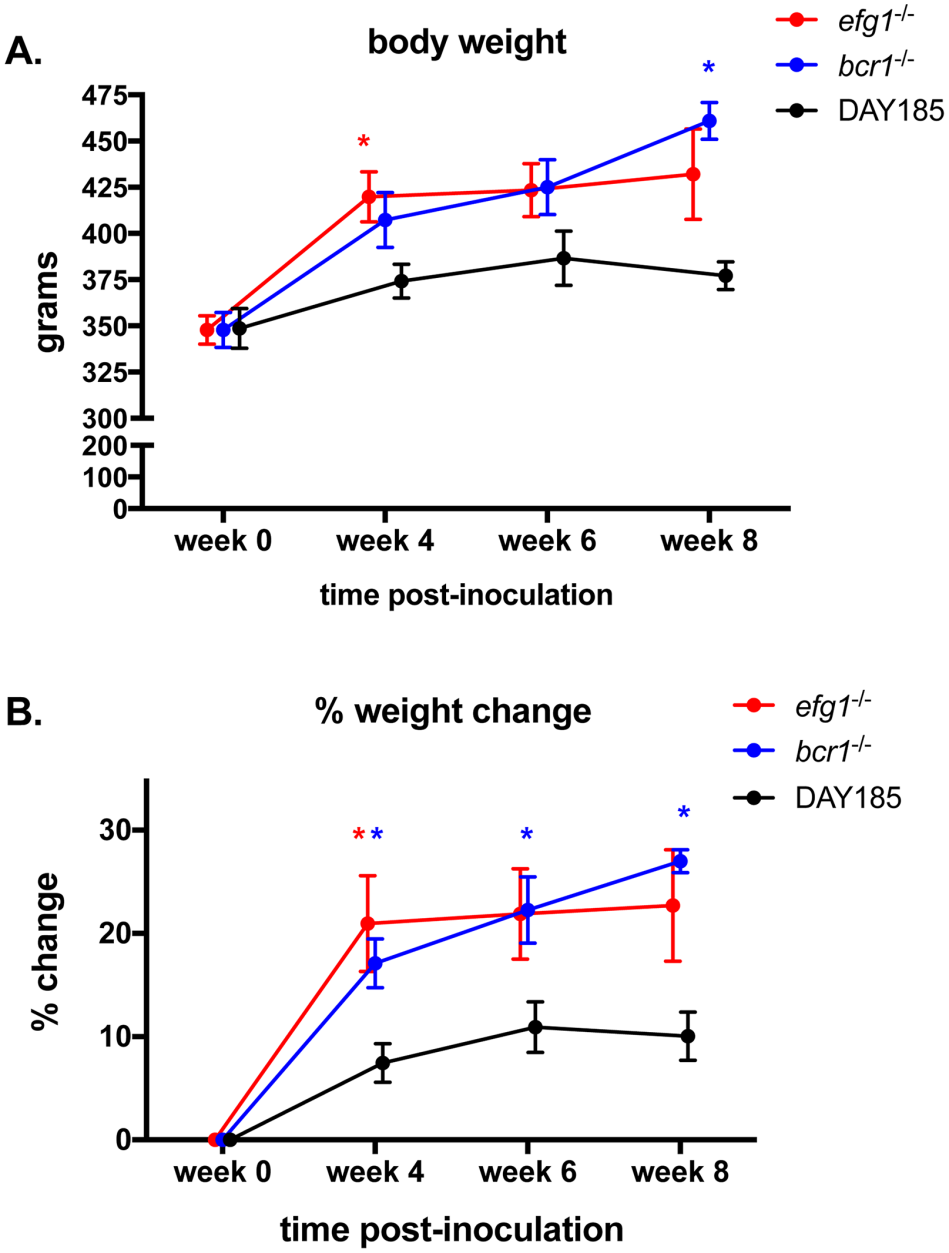
Body weight change over time in rats fitted with dentures inoculated with *C*. *albicans* biofilm deficient strains. Equilibrated rats were fitted with dentures, weaned onto gel diet, given antibiotics in the drinking water, and inoculated 3x with 1 x 10^9^ *C*. *albicans* DAY185, *efg1*^*-/-*^ or *bcr1*^*-/-*^. Rats were weighed bi-weekly for a period of 8 weeks post-inoculation using 3–4 animals per group and data are shown as (A) absolute weights and (B) % weight change (% weight change = weight at time point/weight at week 0 prior to inoculation). Data for individual weight changes per group were analyzed using the Repeated Measures ANOVA. Weight changes between groups at specific time points (experimental vs. control, actual weight or % change) were analyzed by the unpaired Student’s *t* test. * *P* < 0.05.

To quantitatively assess the role for transcription factors required for biofilm formation in virulence during DS, we measured LDH levels in saliva from infected rats, an indicator of tissue damage. Significantly higher levels of LDH were detected in animals infected with WT strain DAY185, Efg1-reconstituted and Bcr1-reconstituted mutants elicited compared to naïve control animals (P = 0.0233, P = 0.0027 and P = 0.0027, respectively). In contrast, animals infected with the efg1^-/-^ and bcr1^-/-^ strains showed significant reductions in LDH compared to the WT controls, but not different from the naïve controls (Fig 7). Another indicator of virulence is reduced weight gain in rats, possibly due to discomfort in eating from tissue damage in the oral cavity. Rats inoculated with WT strain DAY185 gained relatively little weight, remaining near starting weights over the eight week time period (Fig 8A). In contrast, rats inoculated with *efg1*^-/-^ and *bcr1*^-/-^ mutants steadily gained between 50–100 g, by 8 weeks post-inoculation, corresponding to 20–25% increases (Fig 8A and 8B). These trends in weight gain were similar to uninoculated control rats with dentures at week 4 (data not shown). Similar trends in sub-optimal weight gain were observed in reconstituted mutants up to week 4 post-inoculation with some weight loss (data not shown); however, rats inoculated with reconstituted mutant strains were not analyzed at later time points for full comparison to those inoculated with the mutant stains. Rats inoculated with the WT strain had significantly lower weights compared to those inoculated with the mutant strains at several time points over the observation continuum.

## Discussion

One major hurdle in investigating the role of biofilm formation in DS and other mucosal infections is that many *C*. *albicans* mutants that are deficient in biofilm formation are also deficient in several other virulence traits. Biofilm formation is controlled by a transcriptional network composed of six transcriptional regulators, including Efg1 and Bcr1 [32]. Efg1 is one of several master transcriptional regulators that controls morphogenesis along with other virulence factors not directly involved in filamentation, such as adhesins and degradative enzymes, which also contribute to pathogenesis [33, 34]. It is therefore difficult to separate the role of morphogenesis, biofilm formation, and the co-regulated virulence factors. Bcr1 (Biofilm and Cell Wall Regulator) is a transcriptional factor that more specifically controls biofilm formation via regulation of hyphal adhesins, but is not required for hyphal growth [26, 32, 35]. The defect in biofilm formation is likely due to a lack of proper adhesin expression on the cell surface, but not due to a defect in morphogenesis [26].

Using an established rat model of *Candida*-associated denture stomatitis, we show that rats inoculated with *C*. *albicans* mutants defective in biofilm formation by deletion of the transcription factors, *EFG1* or *BCR1*, have reduced oral tissue damage (via LDH) and normal weight gain compared to the WT and reconstituted strains, indicating defects in virulence. In the case of the *efg1*^-/-^ mutant, but not the *bcr1*^-/-^ mutant, the reduced tissue damage and normal weight gain in the rats was concomitant with reduced colonization of the denture and palate. Because the *bcr1*^-/-^ mutant retained the ability to colonize, this suggests that *BCR1* (and biofilm formation) is also required for virulence independent of simply a defect in adherence. Compared with planktonic *C*. *albicans*, biofilms have been shown to preferentially induce production of cytokines driving Th1 or Th17 responses [20, 36]. In the case of DS, it is possible that a *C*. *albicans* biofilm formed on the denture continually inoculates the host mucosa leading to tissue-associated biofilm formation. The pro-inflammatory nature of the mucosal biofilm could lead to chronic inflammation that fails to clear the infection due to the presence of the contaminated oral device.

Previous studies aiming to examine the role of *C*. *albicans* biofilm formation during oral infection in vivo have focused on oropharyngeal candidiasis (OPC; oral thrush), an infection of immunocompromised patients. Acute pseudomembranous candidiasis is the most often studied form of OPC, presenting as white curd-like lesions on the tongue and oral mucosa, associated with invasion of the epithelium. In contrast, candidal invasion of the palatal tissue during DS is extremely rare, with few yeast cells found in mucosal smears [37, 38]. Animal models of OPC rely on corticosteroid treatment to promote colonization and lesion formation, effectively subduing both innate and adaptive immunity. These models have demonstrated that *BCR1* is required for colonization of the tongue, with reduced lesion surface area and thickness (reduced epithelial invasion) [21]. Using an immunosuppressed rat denture biofim model, the *bcr1*^*-/-*^ mutant also exhibited reduced colonization of the denture material and lack of biofilm formation, again indicating that adhesins are critical for surface adherence [19, 21, 32]. Surprisingly, our studies demonstrated that *BCR1* is not required for optimal colonization of the denture or palate tissue in immunocompetent animals. The disparity may be partially explained by technical differences between our denture stomatitis model and the denture biofilm model. In our denture stomatitis model, rat dentures are custom-fitted which allows optimal contact between the denture and palate tissue [25]. With the denture biofilm model, denture material is fabricated with a small channel between the denture and palate tissue to provide an opening for inoculation of the denture (denture is not removable for inoculation) [19]. There is the possibility that the contact with the palate tissue and/or secreted factors produced by this mucous membrane contributes to retention or growth of the *bcr1*^*-/-*^ mutant in our denture stomatitis model. In addition, salivary flow into or through the channel could promote loss of the bcr1^-/-^ mutant in the denture biofilm model. Another possibility is that with longitudinal sampling, the total fungal burden is not being evaluated. However, similar sampling techniques were used with all *C*. *albicans* strains, and reduced burdens were observed with the efg1^-/-^ mutant, indicating that the sampling technique is sensitive enough to detect changes in fungal burden at the log scale. Further, we demonstrated a role for *BCR1* in promoting pathogenesis of DS in vivo based on host readouts (LDH) as opposed to fungal burden. In agreement with these observations, bcr1^-/-^ elicited less LDH release in a three-dimensional cultured oral mucosa model [21]. Future experiments can include examination of gene expression in these strains to determine which downstream targets are important for virulence during DS as has been examined for OPC [39].

Studies using the murine OPC model have demonstrated that *EFG1* is required for optimal colonization of oral tissues [34], supporting our observation for colonization of both dentures and palate tissue in immunocompetent animals. We also demonstrated that *EFG1* is required for both morphogenesis and biofilm formation on dentures and palate tissue in vivo. Reduced colonization correlated with normal weight gain and significantly reduced tissue damage in the oral cavity, supporting previous observations in vitro using reconstituted human epithelium [40]. Interestingly, we observed a partial phenotype with the reconstituted strain in terms of biofilm formation on the palate. Previous studies with this strain have also demonstrated intermediate phenotypes with *EFG1* reconstituted strains in systemic infection models [34]. This could be due to a gene dosage effect, although there is the possibility that the *efg1*^-/-^ strain carries a secondary mutation that affects biofilm formation. Regardless, our reconstituted strain elicited similar LDH levels and stunted weight gain as the wildtype strain, indicating that the partial phenotype did not reduce virulence

The role of key regulators of morphogenesis and biofilm formation has also been examined in a murine model of vulvovaginal candidiasis (VVC). In this model, immunocompetent female estrogenized mice are inoculated intravaginally and hallmark inflammatory responses are monitored at early time points (days). Similar to our results in the DS model, there was a crucial role for *EFG1*, and to a lesser extent, the *BCR1* pathways in contributing to vaginitis immunopathology, including tissue damage as well as biofilm formation [29, 41]. However, in the VVC model the *efg1*^-/-^ mutant exhibited comparable colonization levels to the wildtype strain [41]. The reason for this discrepancy is likely due to the time frame of the infections; day 3 in VVC as an acute infection model, and weeks 4–8 in DS as a chronic infection model. Similar trends were also observed with a *efg1*^-/-^ mutant in a GI tract colonization model, which had an initial advantage during colonization but was preferentially lost over time and became less fit than a WT strain by day 18 [42].

While DS and VVC are both infections that occur in otherwise healthy individuals, there are notable differences in pathogenesis. Onset is acute/recurrent with VVC and chronic with DS. Common symptoms with VVC include presence of discharge, and severe itching and burning, while patients with DS are often asymptomatic despite the presence of clinical signs of erythema and papillary hyperplasia. These differences are likely due to physiological and/or immunological differences in the mucosal tissues (oral cavity vs. vagina). Despite this, Efg1 and Bcr1 are key factors for virulence, indicating similar genes and pathways are involved in stimulating inflammatory responses in both mucosal infections. Results for the *bcr1*^-/-^ mutant showing comparable denture and palate colonization to the control strains, along with reduced tissue damage, suggests a clear role of BCR1 in the pathogenesis of DS. For the *efg1*^-/-^ mutant the results are less clear because reduced tissue damage was concomitant with reduced colonization of the denture and palate. This more severe phenotype is likely due to the fact that Efg1 binds to (and presumably regulates) more target genes than Bcr1 during biofilm formation (328 vs 211, respectively) [32].

Overall, DS represents an excellent infection model to better understand the role of *Candida* monomicrobial and polymicrobial biofilms, and factors promoting biofilm formation, in disease pathogenesis. In addition, because DS affects immunocompetent individuals, future studies can focus on how the immune response plays a role in either protection against or exacerbation of disease, which would fill a large gap in knowledge for a highly prevalent but understudied form of candidiasis. While DS is often asymptomatic, there are other potential negative sequelae possible resulting from constant exposure to a contaminated denture, and has very high recurrence rates despite treatment with antifungal therapy [15, 43]. For example, angular chelitis and OPC have been reported as possible infectious sequelae, with similar species and strains isolated in both diseases [44, 45]. Chronic oral infection could also lead to seeding of the GI tract, which serves as a major portal for systemic infection in immunosuppressed or hospitalized patients. In fact, DS patients have higher rates of GI tract carriage of *Candida*, with similar species isolated from the oral cavity and feces [14]. This could become clinically important in the elderly population, which have high rates of denture wearing and increased risk of developing immunosuppressive diseases. Future studies using the DS model can focus on the effects of subsequent immunosuppression on GI tract colonization and/or dissemination.
